# A New Simulation Method to Assess Temperature and Radiation Effects on SiC Resonant-Converter Reliability

**DOI:** 10.3390/ma19020228

**Published:** 2026-01-06

**Authors:** Zhuowen Feng, Pengyu Lai, Abu Shahir Md Khalid Hasan, Fuad Fatani, Alborz Alaeddini, Liling Huang, Zhong Chen, Qiliang Li

**Affiliations:** 1 Department of Electrical Engineering, University of Arkansas, Fayetteville, AR 72701, USA; zhuowenf@uark.edu (Z.F.); plai@uark.edu (P.L.);; 2 Department of Electrical and Computer Engineering, George Mason University, Fairfax, VA 22030, USAlhuang20@gmu.edu (L.H.); 3 Department of Advanced Manufacturing and Robotics, Peking University, Beijing 100871, China

**Keywords:** silicon carbide power devices, gate oxide degradation, temperature-sensitive electrical parameters (TSEPs), resonant converter, total ionizing doses (TIDs)

## Abstract

Silicon carbide (SiC) power converters are increasingly used in automotive, renewable energy, and industrial applications. While reliability assessments are typically performed at either the device or system level, an integrative approach that simultaneously evaluates both levels remains underexplored. This article presents a novel system-level simulation method with two strategies to evaluate the reliability of power devices and a resonant converter under varying temperatures and total ionizing doses (TIDs). Temperature-sensitive electrical parameters (TSEPs), such as on-state resistance ($R_{\mathrm{ON}}$) and threshold voltage shift ($\Delta V_{\mathrm{TH}}$), are calibrated and analyzed using a B1505A curve tracer. These parameters are incorporated into the system-level simulation of a 300 W resonant converter with a boosting cell. Both Silicon (Si) and SiC-based power resonant converters are assessed for power application in space engineering and harsh environments. Additionally, gate-oxide degradation and $\Delta V_{\mathrm{TH}}$-related issues are discussed based on the simulation results. The thermal-strategy results indicate that SiC MOSFETs maintain a more stable conduction loss at elevated temperatures, exhibiting higher reliability due to their high thermal conductivity. Conversely, increased TIDs result in a negative shift in conduction losses across all SiC devices under the radiation strategy, affecting the long-term reliability of the power converter.

## 1. Introduction

**Figure 1:**
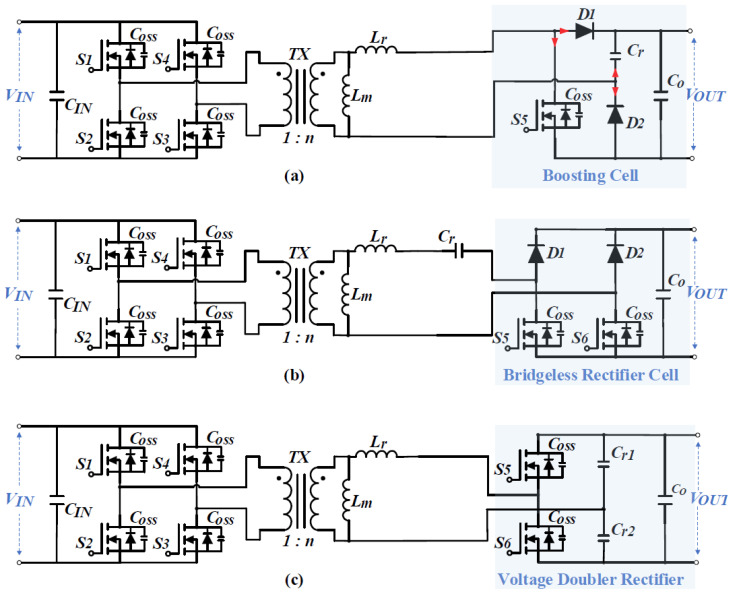
Three types of resonant converters with a boost rectifier cell: (**a**) boosting rectifier cell with only one switch and two diodes [12]; (**b**) bridgeless rectifier cell with two switches and two diodes [9]; (**c**) voltage doubler with two switches [10].

Silicon carbide (SiC) is a representative wide-bandgap semiconductor that supports a broad range of applications, including quantum technologies, luminescent nanostructures, ultra-fine fibers, and implantable biomedical devices [1,2,3,4]. The existence of multiple SiC polytypes with distinct stacking sequences leads to different physical properties. In particular, 4H-SiC, a common polytype of SiC, offers a high critical electric field and high thermal conductivity [5]. In the past decade, DC-DC power converters have gained significant attention due to their expanding role across sectors ranging from aerospace to renewable energy. In renewable energy systems, especially grid-tied photovoltaic applications galvanically isolated, unidirectional LLC (Inductor–Inductor–Capacitor) resonant converters have become common and the focus of numerous studies [6]. Efforts in low-voltage power converters have concentrated on improving key characteristics such as reliability, wide input and load ranges, low electromagnetic interference (EMI), and high efficiency. According to Parvez, Mohammad et al. [7], isolated resonant converters can be broadly classified into three types: resonant-boost converters, hybrid resonant converters, and resonant single-ended primary-inductor converters (SEPICs). Resonant-boost converters are commonly employed in solar-energy systems to accommodate fluctuations in solar input. Recent advancements have introduced hybrid resonant converters with improved rectifier cells, offering a broad input-voltage range and California Energy Commission (CEC) efficiencies exceeding 96% [8,9,10,11,12]. These topologies are designed to minimize component counts in rectifier tanks and extend the regulation range, as shown in Figure 1. For example, a resonant-boost converter with a simplified boosting rectifier cell has been proposed [12]. While this design reduces voltage stress on the resonant capacitor by clamping it at the output-voltage level, it does not fully accommodate variations in input voltage and output power. Although this topology has a limited input-voltage regulation range due to high step-up ratios and load currents [13], it remains valuable for studying the switching performance and reliability of modern power devices.

Recently, SiC power device degradation has been extensively discussed at the device level under harsh environments such as high temperature and radiation [14,15,16,17]. However, the reliability of power converters, especially under high radiation doses, remains underexplored. Existing reliability assessments for power converters mostly rely on experimental testing, which is costly and case-specific. Thus, the development of system-level evaluation frameworks that explicitly incorporate device degradation is needed. Although several simulation-based SiC DC-DC converters have been designed and evaluated using the PSIM manufacturer simulation tool [18,19,20,21], and while these studies analyze power losses and overall efficiency, they lack in-depth analyses of device reliability and do not fully leverage the simulation tool’s capabilities. For system-level simulation of power converters, physics-based models often suffer from convergence issues and long runtimes [22,23,24], whereas simplified models in simulation tools such as PSIM or PLECS sacrifice accuracy. To achieve both computational efficiency and degradation-aware modeling, a methodology that explicitly incorporates degradation effects in SiC power MOSFETs is required.

In this study, a novel simulation framework to model thermal- and radiation-induced degradation and quantify its impact on power converter efficiency is developed. This approach facilitates extensive research without being constrained by datasheet limitations. Also, these two strategies offer a convenient approach for researchers and application engineers to evaluate the reliability of power converters under harsh environment conditions before conducting experiments. For comparison, both Si- and SiC-based resonant converters with a boost cell are evaluated in PSIM. In addition, the results show that SiC-based converters are more reliable than their Si-based counterparts in harsh environments.

## 2. New Proposed Simulation Methodology

### 2.1. PSIM Thermal Model

Dynamic simulation in complex converter topologies remains a challenge for achieving a balance between accuracy, convergence reliability, and simulation speed [25]. For new design topologies, the ideal models are first employed to verify the feasibility of the topology and its control scheme. Additionally, the thermal model can be employed to estimate switching losses and conduction losses in power devices. In PSIM [26], dedicated thermal models idealize switching transitions and evaluate losses using look-up tables. The parameters for the look-up table used in this work are shown in Table 1. The calculations in the PSIM simulation can account for various conditions, such as the conducted voltage and current across the switch, junction temperature and gate drive voltage, and physic parameters such as parasitic capacitance and $R_{\mathrm{ON}}$. It provides a good correlation with actual power losses while significantly reducing the computational time compared to the SPICE model. Although the present thermal model does not account for device degradation, degradation-aware device models can be incorporated in the future to enable system-level performance analysis. From both reliability and lifetime perspectives [27,28], this integration offers clear benefits. It enables more predictive loss and stress estimation and reduces reliance on costly long-duration experiments.

**Figure 2:**
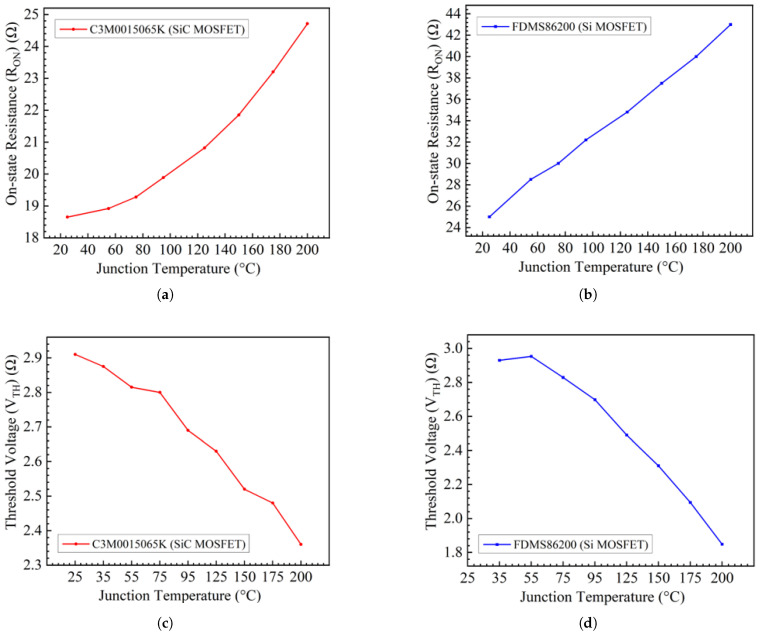
(**a**) SiC MOSFET (C3M0015065K) junction temperature vs. $R_{\mathrm{ON}}$; (**b**) Si MOSFET (FDMS86200) junction temperature vs. $R_{\mathrm{ON}}$; (**c**) SiC MOSFET (C3M0015065K) junction temperature vs. $V_{\mathrm{TH}}$; (**d**) Si MOSFET (FDMS86200) junction temperature vs. $V_{\mathrm{TH}}$.

In PSIM, the calculation of conduction losses ($P_{\mathrm{cond}}$) and on/off switching losses ($P_{\mathrm{sw},\mathrm{on}/\mathrm{off}}$) is given by [21]

(1) $$P_{\mathrm{cond}}=I_{\mathrm{RMS}}^{2}\;R_{\mathrm{ON}}$$

(2) $$P_{\mathrm{sw},\mathrm{on}/\mathrm{off}}=E_{\mathrm{on}/\mathrm{off}}\;f_{\mathrm{sw}}$$

where $I_{\mathrm{RMS}}$ is the root-mean-square value of the conducted current (obtained from the switching waveform) and $R_{\mathrm{ON}}$, which depends on the junction temperature $T_{J}$. $R_{\mathrm{ON}}$ can be expressed as

(3) $$R_{\mathrm{ON}}=R_{\mathrm{ON},\mathrm{Norm}}\;\left[\;1+K_{T}\;\left(T_{j}-25\;{}^{\circ}C\right)\;\right]$$

where $R_{\mathrm{ON},\mathrm{Norm}}$ is the normalized on-state resistance at 25°C (from the datasheet’s normalized $R_{\mathrm{ON}}$ versus temperature curve) and $K_{T}$ is the corresponding temperature coefficient. However, in the thermal strategy, $K_{T}$ is not applied in the simulation because the actual $R_{\mathrm{ON}}$ is obtained directly using a curve tracer. The junction temperature is fixed at 25°C, so the measured $R_{\mathrm{ON}}$ and $V_{\mathrm{TH}}$ from Figure 2 can be used in the thermal model without calculating $K_{T}$, providing a flexible and practical solution for system evaluation.

### 2.2. A New Simulation Method

**Figure 3:**
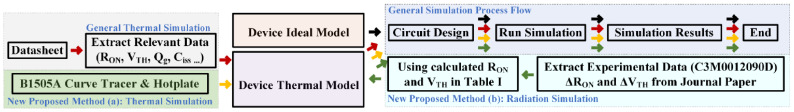
Proposed simulation methodology with two strategies: thermal simulation and radiation simulation.

A newly proposed simulation method to assess the efficiency and reliability of the DC-DC resonant converter includes two strategies: (a) thermal simulation and (b) radiation simulation as illustrated in Figure 3. Both simulations utilize the PSIM simulation tool and its thermal model. In this paper, the SiC-based resonant converter is evaluated under a wide-range temperature and radiation dose conditions. The conventional simulation approach employs a thermal model to estimate the switching and conduction losses for each device in the system. This model is based on datasheet characteristics, which can limit the exploration and evaluation of device performance in system-level analysis, especially for scenarios such as a resonant converter operating at high temperatures exceeding 175 °C.

To analyze the performance and degradation of SiC devices in a resonant tank, a simulation method is illustrated in Figure 3. This method includes two key strategies:

1. Thermal Strategy: This involves calibrating the power device using the B1505A curve tracer (Keysight, Santa Rosa, CA, USA), with the device placed on a hot plate for high-temperature testing. The resulting characteristics, typically not provided in the datasheet, are then applied in thermal simulation using a system-level simulation tool.
2. Radiation Strategy: This utilizes relevant data, specifically the changes in $\Delta R_{\mathrm{ON}}$ and $\Delta V_{\mathrm{TH}}$, from the article [14] on the commercial SiC power device (C3M012090D) (Wolfspeed, Durham, NC, USA) under investigation. The $R_{\mathrm{ON}}$ and $V_{\mathrm{TH}}$ values calculated at various total ionizing doses (TIDs), as shown in Table 2, are then integrated into the PSIM thermal model.

It is worth noting that PSIM-based thermal simulations have been adopted in prior converter-level studies, where simulation results were experimentally verified and showed good agreement with measured data [29,30,31]. In addition, the device degradation considered in this work is obtained from experimentally measured data reported in the literature as well as B1505A-based device characterization. Although this study does not include experimental validation, the simulation results exhibit good consistency with estimates obtained from widely accepted analytical loss models [21], supporting the physical plausibility of the observed trends.

Both thermal and radiation strategies offer valuable benefits for researchers engaged in extensive studies. While this work focuses on analyzing only the $R_{\mathrm{ON}}$ and $V_{\mathrm{TH}}$ parameters at the device level, other parameters listed in Table 1 can also be modified and examined. This approach enables device-level degradation insights to be extended to system-level analysis. Although current system-level simulators are limited and power loss remains the principal observable of the converter after device degradation, the framework developed here is readily extensible. The present method cannot yet resolve degradation effects at the control-waveform level. In the future, degradation-aware device models could relate device degradation to system-level dynamics, for example, by accounting for BTI (Bias Temperature Instability)-induced shifts in the gate plateau that delay rising and falling edges, and threshold voltage variation that affects the voltage slew rate [32,33,34]. In subsequent studies, false turn-on events could become observable under high slew-rate and crosstalk conditions, especially in the presence of both aging-induced and radiation-induced threshold shifts.

### 2.3. Device Calibration

**Figure 4:**
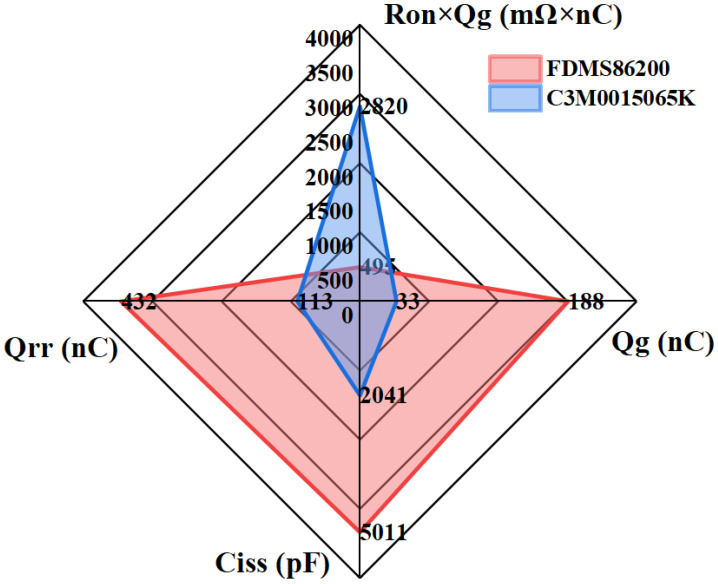
Radar chart comparison of key parameters between Si MOSFETs (FDMS86200) and SiC MOSFETs (C3M0015065K).

**Figure 5:**
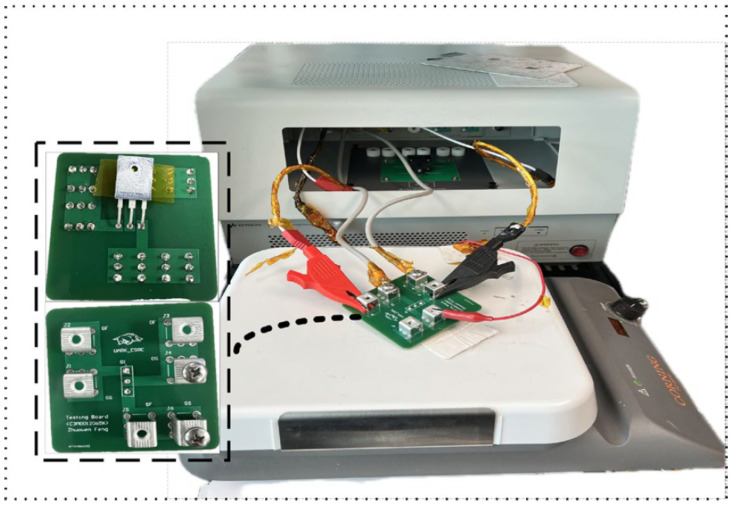
Experimental setup for device calibration on a hot plate for temperature-dependent characterization.

This study examines two commercial devices, a Si MOSFET (FDMS86200) (Onsemi, Scottsdale, AZ, USA) and a SiC MOSFET (C3M0015065K) (Wolfspeed, Durham, NC, USA), each with a typical $R_{\mathrm{ON}}$ of 15 mΩ. Table 1 summarizes the key device parameters, and Figure 4 compares their dynamic performance and figure of merit (FOM). To address potential discrepancies between datasheet transfer characteristics and actual device behavior, the B1505A curve tracer was used for precise measurement of $R_{\mathrm{ON}}$ and $V_{\mathrm{TH}}$. The combination of an extended printed circuit board (PCB) and hot plate to control the junction temperature is illustrated in Figure 5. During testing, the device under test (DUT) was placed on a hot plate for 15 min to allow the junction temperature to stabilize at the target level. Also, other quasi-static parameters can be characterized as a function of temperature using the same setup, enabling quantitative assessment of temperature-accelerated degradation.

The methodology for extracting $R_{\mathrm{ON}}$ and $V_{\mathrm{TH}}$, as described in [35], was applied at different temperatures in this work, with the results shown in Figure 2. The SiC MOSFET exhibited less variation in $R_{\mathrm{ON}}$ compared to the Si MOSFETs, which can be attributed to the thermal conductivity of the SiC. In fact, it is worth noting that the radiation strategy also employs the B1505A for device calibration prior to irradiation [14]. Moreover, a measurement setup similar to the temperature-accelerated degradation calibration can be used to characterize total ionizing dose (TID) effects, enabling assessment of both static and dynamic degradation [36].

### 2.4. Radiation Simulation

In radiation strategy, experimental data is extracted and calculated from [14] due to the lack of a radiation source. This approach provides valuable insights for application engineers to analyze device performance in harsh environments, which is discussed in detail below.

#### 2.4.1. Physical Mechanism of SiC MOSFET Degradation

**Figure 6:**
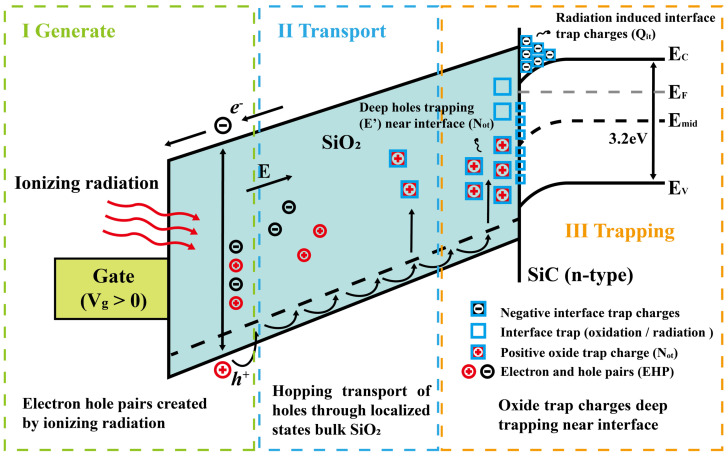
Energy band diagram of radiation-induced charge dynamics in the SiO_2_/SiC system under positive gate bias.

As previously mentioned, the $R_{\mathrm{ON}}$ of SiC MOSFETs mainly depends on channel resistance, which is influenced by mobility and $V_{\mathrm{TH}}$. When a SiC MOSFET is exposed to gamma radiation, electron–hole pairs are generated in the gate oxide. Due to their high mobility, electrons are more likely to escape from the gate oxide, while holes become trapped within the gate oxide, leading to an accumulation of negative charges at the SiO_2_/SiC interface. Thus, the accumulation of oxide trap charges following irradiation leads to greater inversion in the NMOS channel and a negative shift in the threshold voltage. The mechanism of threshold voltage degradation under radiation doses in SiC MOSFETs is illustrated in Figure 6.

To describe the TID irradiation-induced degradation of $V_{\mathrm{TH}}$, a model derived by [14] can be applied:

(4) $$\Delta V_{\mathrm{TH}}=-\frac{qN_{h}T_{\mathrm{ox}}^{2}f\left(t\right)}{\varepsilon_{\mathrm{ox}}}\times f\left(E\right)\times \mathrm{Dose}$$

where *q* is the elementary charge, $N_{h}$ is the number of generated electron–hole pairs, $T_{\mathrm{ox}}$ is the gate-oxide thickness, f(t) is the fraction of positive oxide trap charges, $\varepsilon_{\mathrm{ox}}$ is the dielectric constant of oxide, f(E) is the hole yield as a function of the electric field, and *Dose* is the total ionizing dose in rads.

Under different bias conditions, the trapping effect results in varying degradation behavior due to the electric field. When the MOSFET is on, it operates under a gate bias condition; when off, it operates under drain bias. Under gate bias, a high positive electric field is applied in the gate oxide above the channel region, while under drain bias, a comparatively lower negative electric field is present above the near-channel region. As a result, SiC MOSFETs exhibit greater sensitivity to gate bias during γ-ray irradiation, particularly in terms of changes in $V_{\mathrm{TH}}$ and $R_{\mathrm{ON}}$. In the simulation, SiC MOSFETs (C3M012090D) are used in [14]. The extracted $\Delta R_{\mathrm{ON}}$ and $\Delta V_{\mathrm{TH}}$ under gate bias, as shown in Table 2, are applied in the simulation.

In Equation (4), $\Delta V_{\mathrm{TH}}$ is proportional to the TIDs, the electric field in the gate oxide, and the oxide thickness. Given that the oxide layer in SiC MOSFETs is typically thicker than 45 nm, electrons have sufficient space to accelerate under the electric field, gaining more than 8.9 eV of energy. This energy allows them to collide with SiO_2_ lattice atoms, generating electron–hole pairs [37]. Due to the oxide thickness, $V_{\mathrm{TH}}$ variation in planar SiC MOSFET is significantly smaller than in trench SiC MOSFETs at higher dose levels [38,39]. However, limited research compares planar-gate and split-gate MOSFETs under high radiation doses. Based on Equation (4), it can be inferred that the $V_{\mathrm{TH}}$ variation in split-gate MOSFETs might be significantly smaller than that in planar MOSFETs, as the electric field in the gate oxide is much lower in split-gate designs.

#### 2.4.2. Threshold Voltage Shift Induced by Oxide Traps

The $V_{\mathrm{TH}}$ of SiC MOSFETs can be affected by radiation-induced oxide traps. The sensitivity of $V_{\mathrm{TH}}$ depends on TIDs and the electric field generated by the trapped hole. In the experiment [14], a negative shift in $V_{\mathrm{TH}}$ and $R_{\mathrm{ON}}$ was observed under γ-ray irradiation. For worst-case assessment, the $V_{\mathrm{TH}}$ shift caused by radiation-induced trapped charges should be evaluated together with the $V_{\mathrm{TH}}$ shift induced by high-temperature gate bias (HTGB) under negative gate bias [40].

When HTGB and TIDs are simultaneously applied, Zhang et al. [41] observes a substantial shift in $V_{\mathrm{TH}}$. Therefore, although the $R_{\mathrm{ON}}$ decreases under TIDs, the overall power consumption of power devices in harsh environments (with both high temperature and high radiation doses) increases, potentially reducing the efficiency of power applications. Unfortunately, limited research addresses the performance of power devices at the application level under harsh environments, including online $R_{\mathrm{ON}}$ measurements. Consequently, by experimentally characterizing parameter drifts under concurrent BTI and TID and integrating the extracted models into the proposed framework, the system-level impact on efficiency can be evaluated by using the proposed method.

## 3. Isolated Series Resonant Converter

In this paper, a galvanically isolated series resonant converter (SRC) topology is selected and designed. A brief introduction to the resonant converter is provided below.

### 3.1. Resonant Boost Converter Topology

The converter features a front-end full bridge and a boosting cell, connected through a galvanically isolated transformer [12]. It offers significant advantages, including reduced stress on the resonant capacitor, which regulates the output voltage, as well as a lower component count compared to the topologies in Figure 1. The proposed converter can operate in four distinct modes, all based on a discontinuous resonant current with a quality factor of less than one, irrespective of load conditions. The pure-SRC mode is employed solely in the simulation. The prototype developed for this study has a power rating of 300 W and achieves an output voltage of 355 V and the design parameters of the proposed converter are included in Table 3.

As a wide-input-range DC–DC resonant converter, this type is particularly suited for solar applications, especially in space solar applications. Therefore, it is essential to evaluate the performance of the converter in harsh environments, such as high temperature and high radiation doses. In this paper, two strategies are proposed and implemented to simulate the power losses of the converter under these extreme conditions.

### 3.2. Zero-Voltage Switching

To minimize turn-on switching losses, zero-voltage switching (ZVS) has been implemented in this topology. As a result, turn-on switching loss is negligible, and the turn-off loss is determined by the fall time. The turn-off voltage slew rate of the device is influenced by the gate drive circuit design.

**Figure 7:**
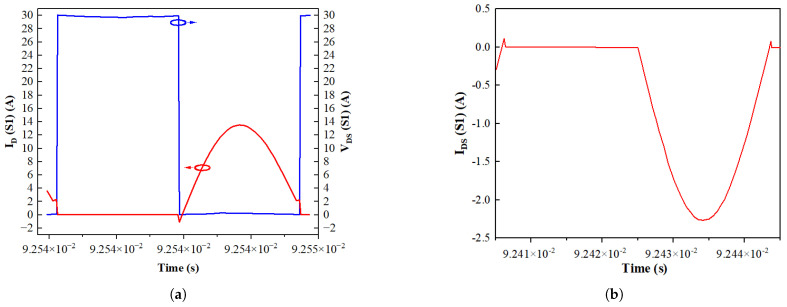
(**a**) $V_{\mathrm{DS}}$ (green) and $I_{D}$ (red) of S1 showing the implementation of ZVS during dead time; (**b**) drain current $I_{D}$ of S5 showing the implementation of ZCS during dead time.

In the front-end full bridge, the transformer’s magnetizing inductance ($L_{m}$) serves as the current source during dead time. Thus, the magnetizing inductance should be designed such that the magnetizing current can fully charge and discharge the parasitic output capacitance of S1–S4, facilitating ZVS [12]. Figure 7 shows the drain–source voltage ($V_{\mathrm{DS}}$) and drain current of S1 over one period, clearly demonstrating ZVS in this design. However, it is important that the magnetizing inductance of the transformer be designed according to the following criterion:

(5) $$L_{m}\leq \frac{n^{2}T_{\mathrm{DT}}}{8f_{\mathrm{sw}}C_{\mathrm{OSS}}},$$

where *n* is the transformer turns ratio, $T_{\mathrm{DT}}$ is the dead time in the primary-side gate signals, $f_{\mathrm{sw}}$ is the switching frequency of the power devices, and $C_{\mathrm{OSS}}$ is the parasitic output capacitance of the front-end full-bridge switches.

When designing a resonant converter, it is crucial to account for the switch parasitic. In addition to the output capacitance, parameters such as gate charge and the dynamic characteristics of the body diode must align with the circuit design. The proposed strategy provides an effective approach for analyzing how these device parameters interact with the circuit, thereby ensuring optimal performance.

## 4. Simulation Results and Reliability Analysis

### 4.1. Temperature-Sensitive Electrical Parameters (TSEPs)

Due to their excellent material characteristics, SiC MOSFETs can operate at extremely high temperatures and are suitable for harsh environment applications. Various TSEPs of SiC MOSFETs, such as on-state resistance ($R_{\mathrm{ON}}$) and threshold voltage ($V_{\mathrm{TH}}$), should be analyzed.

#### 4.1.1. On-State Resistance ($R_{\mathrm{ON}}$)

Gate-oxide degradation and total $R_{\mathrm{ON}}$ are critical aging indicators for SiC MOSFETs. In both typical planar MOSFET and trench MOSFET shown in Figure 1, the total $R_{\mathrm{ON}}$ is obtained by summing the individual resistances of each layer and region, which can be represented as

(6) $$R_{\mathrm{ON}}=R_{\mathrm{CH}}+R_{\mathrm{JFET}}+R_{A}+R_{D}+R_{N^{+}}+R_{\mathrm{SUB}}+R_{\mathrm{CS}}+R_{\mathrm{CD}}.$$

Here, $R_{\mathrm{CH}}$ is the channel resistance, $R_{N^{+}}$ is the source region resistance, $R_{\mathrm{JFET}}$ is the resistance of the JFET region (Junction Field-Effect Transistor), and $R_{A}$, $R_{D}$, and $R_{\mathrm{SUB}}$ represent the accumulation resistance, the resistance of the drift region, and the $N^{+}$ substrate resistance, respectively. $R_{\mathrm{CS}}$ is the source contact resistance, while $R_{\mathrm{CD}}$ is the drain contact resistance. However, the dominant contributors to $R_{\mathrm{ON}}$ are different between Si MOSFETs and SiC MOSFETs (1.2 kV planar MOSFET). In Si MOSFETs, $R_{\mathrm{JFET}}$ and $R_{D}$ are the primary contributors due to lower breakdown electric-field strength and a thicker drift layer. To achieve a comparable breakdown-voltage rating, the lower critical electric field of Si requires a thicker drift layer to maintain the blocking voltage. In contrast, $R_{\mathrm{CH}}$ is a major contributor to $R_{\mathrm{ON}}$ in SiC MOSFETs due to lower inversion carrier mobility at the SiO_2_/SiC interface [42,43]. Therefore, excluding $R_{\mathrm{CH}}$, the rest of the resistances can be defined as the residual resistance ($R_{S}$). The total $R_{\mathrm{ON}}$ is the sum of $R_{\mathrm{CH}}$ and $R_{S}$, where the contribution of channel resistance $R_{\mathrm{CH}}$ is given by [44]

(7) $$R_{\mathrm{CH}}=\frac{L_{\mathrm{CH}}\;P}{\mu_{\mathrm{inv}}\;C_{\mathrm{OX}}\;\left(V_{G}-V_{\mathrm{TH}}\right)},$$

where $L_{\mathrm{CH}}$ is the channel length, *P* is the pitch of the MOSFET elementary cell, $\mu_{\mathrm{inv}}$ is the electron mobility in the inversion layer, $C_{\mathrm{OX}}$ is the specific gate-oxide capacitance, $V_{G}$ is the applied gate voltage, and $V_{\mathrm{TH}}$ is the threshold voltage.

In [45], it is noted that $R_{\mathrm{JFET}}$ and $R_{D}$ exhibit a positive temperature coefficient (PTC), while $R_{\mathrm{CH}}$ displays a negative temperature coefficient (NTC). As discussed above, $R_{\mathrm{CH}}$ is the primary contributor to $R_{\mathrm{ON}}$ in SiC MOSFETs. As shown in Figure 2, in the temperature range of 25–140 °C, the SiC device shows a relatively smaller increase in $R_{\mathrm{ON}}$ compared with the Si device. This behavior can be attributed to the dominant channel resistance, exhibiting a negative temperature coefficient (NTC) in this range, which partially offsets the positive temperature coefficient (PTC) increase in the residual resistance [46]. Many studies have reported that channel resistance can exhibit an NTC behavior [43,47,48], which is mainly associated with the temperature dependence of the carrier mobility and the effective gate overdrive $\left(V_{G}-V_{\mathrm{TH}}\right)$. For SiC MOSFETs, $V_{\mathrm{TH}}$ is more temperature-sensitive due to the high interface-state density $D_{\mathrm{it}}$ at the SiO_2_/SiC interface [46], which affects the temperature dependence of the effective gate overdrive $\left(V_{G}-V_{\mathrm{TH}}\right)$. As a result, the effective gate overdrive $\left(V_{G}-V_{\mathrm{TH}}\right)$ increases as temperature increases, thereby partially reducing $R_{\mathrm{CH}}$. This NTC behavior of $R_{\mathrm{CH}}$ results in a weaker temperature sensitivity of $R_{\mathrm{ON}}$ in SiC MOSFETs compared with Si MOSFETs over the studied temperature range. At a high temperature up to 375°C, $R_{\mathrm{CH}}$ no longer dominates $R_{\mathrm{ON}}$; instead, $R_{D}$ and $R_{\mathrm{JFET}}$ in $R_{S}$ become more influential as the temperature increases [49]. Overall, the increase in $R_{\mathrm{ON}}$ for the SiC MOSFET remains smaller than that of the Si MOSFET at elevated temperatures in this study.

#### 4.1.2. Threshold Voltage ($V_{\mathrm{TH}}$)

As discussed in Section 2, $V_{\mathrm{TH}}$ is a radiation-sensitive parameter that tends to shift negatively during gamma-ray radiation. However, it is also sensitive to temperature changes. The threshold voltage of the SiC MOSFET can be expressed as follows [49]:

(8) $$V_{\mathrm{TH}}=V_{\mathrm{FB}}+2\Psi_{B}+\frac{1}{C_{\mathrm{OX}}}\left(\sqrt{2\varepsilon_{s}qN_{A}\left(2\Psi_{B}\right)}+q\;\;\int_{E_{i}}^{E_{i}+\Psi_{B}}\;\;D_{it}\left(E\right)\;dE\right)$$

where $\varepsilon_{s}$ is the dielectric constant of SiC, *q* is the electronic charge, $N_{A}$ is the doping concentration of the P-base region, $V_{\mathrm{FB}}$ is the flat-band voltage, $\Psi_{B}$ is the Fermi potential from the substrate, and $D_{it}$ is the interface-state density. Notably, the commercial devices chosen from CREE have shown improvements in the SiO_2_/SiC interface [50]. With the reduction in near-interface traps (NITs), the stability of $V_{\mathrm{TH}}$ performs well under high temperatures. The rate of variation in $V_{\mathrm{TH}}$ with temperature can be expressed as follows [48]:

(9) $$\frac{dV_{\mathrm{TH}}}{dT}=\frac{d\Phi_{ms}}{dT}+\frac{d\Psi_{B}}{dT}\left(2+\frac{1}{C_{\mathrm{OX}}}\sqrt{\frac{\varepsilon_{s}qN_{A}}{\Psi_{B}}}\right)+\frac{q}{C_{\mathrm{OX}}}\;\frac{dN_{it}}{dT}$$

where *T* is temperature, $\Phi_{ms}$ is the work function difference between the metal and the semiconductor at the gate and substrate, and $N_{it}$ is the number of traps between the neutral level and the conduction band. The temperature dependence of $V_{\mathrm{TH}}$ is determined by the $\Psi_{B}$, $\Phi_{ms}$ and $N_{it}$. Specifically, $\Psi_{B}$ decreases with increasing temperature due to the rise in intrinsic carrier concentration. Additionally, $N_{it}$ also decreases with temperature as the Fermi level energy ($E_{f}$) shifts toward the mid-gap, which reduces the occupancy of electrons in the interface traps. In general, the $V_{\mathrm{TH}}$ of SiC MOSFETs shows a negative shift characteristic as the temperature increases.

### 4.2. Thermal Simulation Results

It is important to note that the switching loss for each MOSFET is only an estimate. In the simulation, both the external turn-on and turn-off resistances are set to 1Ω, with other parameters shown in Table 2, which correspond to the switching loss in the PSIM thermal model, as discussed in Section 2.

**Figure 8:**
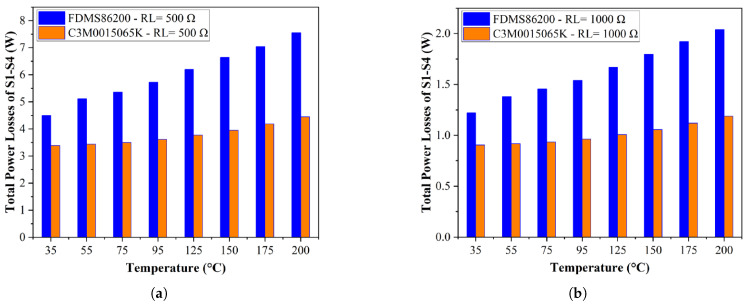
(**a**) Power conduction losses of Si (blue) and SiC (orange) MOSFETs (S1–S4) under different temperatures (R_load_ = 500 Ω). (**b**) Power conduction losses of Si (blue) and SiC (orange) MOSFETs (S1–S4) under different temperatures (R_load_ = 1000 Ω).

In the thermal strategy, the conduction losses of power devices in the front-end full bridge are primarily analyzed to compare the power consumption stability between Si and SiC MOSFETs, which can affect the power converter reliability. As shown in Figure 8, the change in total power conduction losses for SiC MOSFETs is less pronounced than for Si MOSFETs as temperature increases. At a 40.8% load ($R_{\mathrm{load}}=1\;k\Omega$), the variation in $R_{\mathrm{ON}}$ for SiC MOSFETs is only 26%, compared to a significantly higher 67.7% for Si MOSFETs. Similarly, at an 81% load ($R_{\mathrm{load}}=500\;\Omega$), the variation in $R_{\mathrm{ON}}$ for SiC MOSFETs is just 21%, while for Si MOSFETs, it remains significantly higher at 67%.

In summary, the power loss stability of the Si-based resonant converter is lower than that of the SiC-based resonant converter, primarily due to the superior $R_{\mathrm{ON}}$ stability of SiC under high temperatures. However, it is important to emphasize that the switching performance discussed is only an estimate and depends on the gate driver design. As previously noted, the switch’s fall time significantly affects switching performance, especially when achieving ZVS. SiC devices, with their high dv/dt and di/dt characteristics, enable faster turn-on and turn-off speeds compared to Si devices. The key consideration is the trade-off involving overshoot. In low input voltage scenarios, the overshoot voltage remains below the breakdown voltage, reducing potential risks. Therefore, SiC MOSFETs in today’s technology are more adaptable to harsh environments, even in low-power applications.

### 4.3. Radiation Simulation Results and Analysis

**Figure 9:**
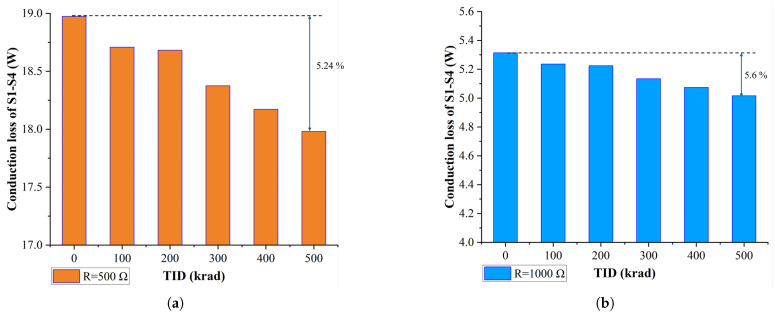
(**a**) Power conduction losses of SiC MOSFETs (S1–S4) under different TIDs (R_load_ = 500 Ω). (**b**) Power conduction losses of SiC MOSFETs (S1–S4) under different TIDs (R_load_ = 1000 Ω).

The radiation simulation results shown in Figure 9 indicate that SiC MOSFETs experience a decrease in $R_{\mathrm{ON}}$ as the TID increases from 0 to 500 krad, with a 5.24% reduction noted at a load resistance of 500Ω. While this reduction in $R_{\mathrm{ON}}$ may initially appear beneficial due to the resulting lower power conduction losses, the threshold voltage shift observed under gamma-ray irradiation raises more significant concerns regarding long-term reliability. A negative shift in $V_{\mathrm{TH}}$ implies that the MOSFET can turn on more easily, which increases the risk of unintended switching in the presence of noise, ultimately compromising circuit performance and reliability. Furthermore, as the load decreases (e.g., at 1000Ω), the overall power conduction losses continue to decrease further, but the impact on $V_{\mathrm{TH}}$ remains considerable. The accumulation of trapped charges in the gate oxide causes a more permanent degradation of $V_{\mathrm{TH}}$, potentially leading to device failure after prolonged operation under radiation. While the decrease in $R_{\mathrm{ON}}$ contributes to lower conduction losses, this trade-off compromises long-term device reliability. In addition, the negative shift in $V_{\mathrm{TH}}$ increases the device’s sensitivity to environmental noise, which poses a significant risk in space applications where long-term reliability is critical.

The combined effects of radiation exposure, high temperature, and prolonged operation must be analyzed in system-level design. A simulation-based method can aid in predicting potential issues that may arise in real experiments. For instance, the permanent degradation of $V_{\mathrm{TH}}$ and the alterations in $R_{\mathrm{ON}}$ could lead to catastrophic device failure, resulting in simulation that fails to accurately replicate real-life conditions.

### 4.4. Future Work

In the future, experimental validation will be pursued to further corroborate the proposed system-level methodology by integrating converter-level measurements with device characterization under elevated temperature or radiation stress, subject to the availability of suitable experimental facilities. In addition, more detailed degradation-aware device models will be incorporated to capture dynamic effects beyond steady-state losses, such as control-waveform distortion, switching transient variation, and gate-plateau shifts induced by device degradation. These extensions aim to enhance the capability of the framework for reliability-oriented system-level analysis under harsh operating conditions.

## 5. Conclusions

This paper proposes a simulation methodology that integrates thermal and radiation strategies within the PSIM thermal-modeling framework. In the thermal strategy, device calibration is performed using a B1505A curve tracer to correct deviations between datasheet-based and measured transfer characteristics. The simulation results indicate that the SiC-based resonant converter exhibits greater power loss stability than its Si-based counterpart at elevated temperatures. In the radiation strategy, the literature-reported experimental data are used to simulate power losses in the resonant converter under the total-ionizing-doses (TIDs) condition. Accordingly, the associated gate-oxide degradation and threshold voltage instability are discussed in this work. Although conduction losses may decrease under certain load conditions, the increase in TIDs is still expected to induce a permanent threshold voltage shift, which reduces the available gate drive operating margin and can adversely affect converter-level robustness. In addition, the absolute magnitude of radiation impact may be underestimated, since other radiation-sensitive effects are not captured in the present model.

## Figures and Tables

**Table 1:** Critical parameters in PSIM thermal model.

Part Number	C3M0015065K	C3M0120090D	FDMS86200
Material	SiC	SiC	Si
$R_{\mathrm{ON}}$ (mΩ)	15	120	15
$V_{\mathrm{DS}}$ (V)	650	900	150
$V_{\mathrm{TH}}$ (V)	2.3	2.1	2.5
$Q_{g}$ (nC)	188	21	33
$Q_{rr}$ (nC)	432	127	113
$C_{\mathrm{iss}}$ (pF)	5011	414	2041

**Table 2:** Calculated $R_{\mathrm{ON}}$ and $V_{\mathrm{TH}}$.

TID (Dose/Krad)	$\Delta R_{\mathrm{ON}}$ (mΩ)	$\Delta V_{\mathrm{TH}}$ (V)	$R_{\mathrm{ON}}$ (mΩ)	$V_{\mathrm{TH}}$ (V)
100	−2.01	−0.24	117.99	1.86
200	−2.21	−0.33	117.79	1.77
300	−4.52	−0.56	115.48	1.54
400	−6.02	−0.71	113.98	1.39
500	−7.44	−0.99	112.56	1.11

**Table 3:** Design parameters of the proposed converter.

Parameter (Symbol)	Value
Input voltage ($V_{\mathrm{in}}$)	10–30 V
Output voltage ($V_{\mathrm{out}}$)	350 V
Switching frequency ($f_{\mathrm{sw}}$)	95 kHz
Resonant capacitor ($C_{r}$)	30 nF
Input capacitor ($C_{\mathrm{in}}$)	150 μF
Output capacitor ($C_{o}$)	150 μF
Resonant inductance ($L_{r}$)	92.5 μH
Magnetizing inductance ($L_{m}$)	1.3 mH
Leakage inductance ($L_{\ell k}$)	4 μH
Transformer turns ratio (1:n)	1:6
Rated output power ($P_{\mathrm{out}}$)	300 W

## Data Availability

The original contributions presented in this study are included in the article. Further inquiries can be directed to the corresponding authors.
